# Unconventional Approaches to Direct Detection of Borreliosis and Other Tick Borne Illnesses: A Path Forward

**DOI:** 10.33696/immunology.3.094

**Published:** 2021

**Authors:** Lance Liotta, Alessandra Luchini

**Affiliations:** George Mason University, Manassas, Virginia, USA

## Direct Molecular Detection, and Not Serology, Can Adjudicate Active Infection

The current COVID-19 pandemic has brought to public attention the conceptual difference between a test for COVID-19 derived RNA or proteins indicating the presence of an active infection, versus COVID-19 serology testing indicating pathogen infection. Only the former test for molecules derived from COVID-19 provides reliable evidence of a current active infection. As such, nucleic acid amplification methods, and COVID-19 antigen immunoassays, are used to diagnose SARS-CoV-2 active infection [1] and possible reinfection [2], to assess infection duration [3] and to guide patient management [4]. COVID-19 serology testing is used only for surveillance purposes [5] (not diagnosis of active infection), to guide public health measures [6], and to monitor vaccine response [7]. Thus, we would not use a positive COVID-19 serum antibody test to indicate the presence of an active COVID-19 infection. In striking contrast, no clinically accredited molecular test exists to detect molecules directly derived from Borreliosis. Nevertheless, treatment decisions concerning the diagnosis of acute and persistent Borreliosis are currently made based on Borreliosis serology testing and clinical evaluation of the patient’s medical history and symptoms [8]. Thus, diagnosis and management of Borreliosis is hampered by subjective tools and indirect markers of the disease. Consequently, there is an urgent need to find, and validate, direct molecular markers derived from the pathogen itself. Integrating a direct test for Borreliosis into clinical practice will dramatically raise the level of evidence-based clinical management for this widespread tick-borne disease. In addition to improved objective diagnosis of active Borreliosis, a direct test can provide important clues about the biologic functional state of the pathogen, leading to insights for pathogenesis and new treatment strategies.

## Borreliosis Infection Signs and Symptoms Can Be Subjective and Multifactorial

Clinical manifestations of Lyme Borreliosis are heterogeneous, depending on different strains of *Borrelia*, possible co-infection with other tick-borne pathogens, and the host response [8]. Erythema migrans, a skin rash that is considered a typical sign of acute infection, varies substantially in shape, color, patterns, and homogeneity, and is influenced by age, sex, skin color, body location, and infection duration [9]. A two-tier serology test is the recommended laboratory method for Borreliosis diagnosis in Europe and in the US [10], with a low diagnostic performance at the disease onset (50% sensitivity) and variable sensitivity at later stages of disease (50%-97%) [10]. PCR testing for *Borrelia*, the only existing direct test, is restricted to synovial fluid for Lyme arthritis and skin biopsy PCR for acrodermatitis chronica atrophicans [10]. Otherwise, PCR has very low, and highly variable sensitivity, depending on the disease stage and type of biospecimen [10]. Objective symptoms associated to disseminated Borreliosis can involve the skin, joints, nervous system, and rarely heart and eyes. Subjective symptoms such as fatigue, myalgia, cognitive complaints may be present at all stages of disease and may persist after treatment completion (Post treatment Lyme disease syndrome). There is general consensus on the natural history and management of patients in the acute phases of Borreliosis. There is NOT consensus, however, on the etiology of long-term post treatment Borreliosis sequelae [10,11]. Widely different theories have been set forth to explain persistence of symptoms in a subset of patients who have gone through a course of antibiotic treatment for Borreliosis. Explanations range from 1) “hit and run” by the pathogen followed by persistent autoimmunity, or residual tissue damage, after the resolution of active infection [12], 2) persistent latent or smoldering infection [13] in systemic tissues and organs distant from the tick bite skin site, to, 3) immune cloaking and immune dysregulation. A novel mechanism for dysregulated immunity, beyond peptide mimicry [14], has been recently proposed [15]. New experimental data suggest that *Borrelia* alters the repertoire of self-peptides bound to MHC II class molecules in professional antigen presenting cells, and induces overexpression of self-antigen presenting MHC II molecules [15]. Authors propose that high levels of MHC II molecule expression above the threshold of established tolerance mechanisms, can induce lymphocyte activation and stimulate an immune response against self-antigens [15]. In parallel, there is also evidence that *Borrelia* can persist in affected tissues in humans for months or years [16]. Animal model studies demonstrated that *Borrelia* induces lymph node architecture disruption, alters normal lymph node T/B cell ratios, prevents the formation of stable germinal centers, and hinders the establishment of long-term memory cell populations [17]. The true nature of persistent Borreliosis in humans will remain unanswered until a direct molecular test of the pathogen itself can be used to objectively provide evidence of the viable organism in symptomatic patients.

## Direct Molecular Testing for Borreliosis Can Settle Important Controversies Concerning Persistent Disease

**Figure 1:**
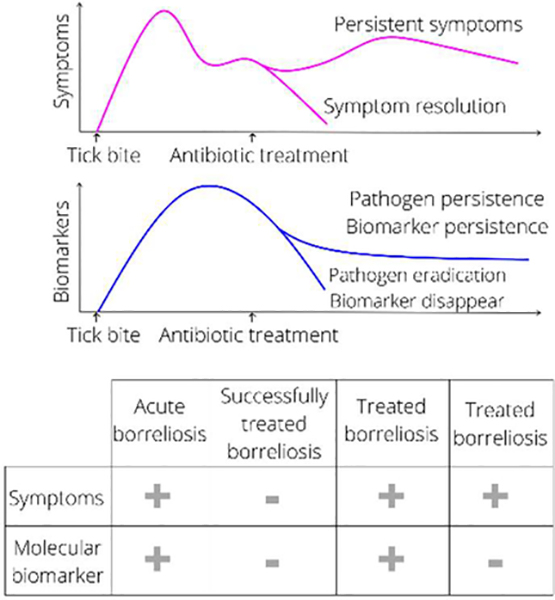
Proposed *Borrelia* biomarker dynamics in relation to clinical symptoms. (Top) Following successful treatment, objective signs and symptoms decline, mirrored by systemic biomarkers. In the case of unsuccessful treatment, symptoms persist and so should peripheral biomarkers, if the pathogen persists. (Bottom) Accurate direct test based on *Borrelia* biomarkers will help differentiate active Borreliosis (+ symptoms, pathogen presence) from inactive disease (+ symptoms, pathogen absence).

Sensitive and accurate molecular direct tests hold the promise to settle the controversies and confirm pathogen presence in treated Borreliosis patients with long term symptoms. A direct test can also be used to diagnose disease before seroconversion, and monitor therapy efficacy (Figure 1).

## Peripheral Fluid Biomarkers for Borreliosis: Proteins Shed by the Pathogen Provide Diagnostic and Functional Information

Unbiased high throughput molecular methods hold the promise to identify pathogens in the face of strain and antigenic variability. Next generation sequencing has been proposed for vector characterization and surveillance studies and has led to new pathogen identification and tick microbiome characterization [18]. The use of next generation sequencing as diagnostic method for pediatric Lyme patients is under clinical evaluation (ClinicalTrials.gov Identifier: NCT03505879). Using a combination of mass spectrometry and immunoassay methods, peptidoglycan persistence was demonstrated in synovial fluid of Lyme arthritis patients [19].

Proteomics can provide functional information on the pathogen and its interaction with the host. We have applied an unbiased proteomics approach to identify tick borne pathogen peptides in the urine of patients under consideration for tick borne illnesses at different stages. In a cohort of 408 cases and controls, we have identified 2 pathogen derived peptides in 9/10 acute EM cases, and 0 false negatives in 250 asymptomatic and symptomatic controls [20]. We found that 40% of PTLDS patients and patients under clinical evaluation for tick borne illnesses had urinary peptides derived from a tick borne pathogen [20]. We identified 160 proteins from *Borrelia* in a cohort of 158 patients under clinical evaluation for tick borne illnesses [20]. Proteins belonged to the following biological pathways: biosynthesis, cell wall organization and biogenesis, cell cycle, chemotaxis, immune evasion, metabolism, signal transduction, transcription/translation, and transmembrane transport.

Peptides derived from 6 enzymes involved in peptidoglycan biosynthesis and degradation were identified (UDP-N-acetylmuramoyl-tripeptide--D-alanyl-D-alanine ligase; UDP-N-acetylmuramate--L-alanine ligase; undecaprenyldiphospho-muramoylpentapeptide beta-N-acetylglucosaminyltransferase; alanine racemase; murein biosynthesis integral membrane protein MurJ; septal ring lytic transglycosylase RlpA family protein). Peptidoglycans are an essential component of *Borrelia* cell wall [19]. *Borrelia* undergoes a peptidoglycan remodeling process to expand its cell wall during bacterial growth [19]. It has been proposed that, in absence of a peptidoglycan recycling system, *Borrelia* sheds 40–50% of its peptidoglycan per generation [19].

## Identifying Functional Weaknesses of *Borrelia*

Due to its small genome, *Borrelia* is deficient in fundamental metabolic pathways such as carbohydrate metabolism, phospholipid metabolism, and fatty acid metabolism. The pathogen therefore relies on the host to obtain its nutrients [21]. Genomic studies demonstrated that carbohydrate metabolism in *Borrelia* is carried on through the glycolysis pathway alone and not through tricarboxylic acid cycle or oxidative phosphorylation [21]. Sole reliance on glycolysis pathway for ATP production might explain *Borrelia* slow growth rate, as *Borrelia* glycolysis yield of ATP is 3 molecules versus 30 molecules in a cell with functioning TCA and OXPHOS [21]. In our proteomics analysis of symptomatic patients, we found *Borrelia* glyceraldehyde-3-phosphate dehydrogenase, which is the sixth step in the glycolysis pathway. It has been shown that *Borrelia* induces human PBMCs to shift to an increased glycolytic activity bypassing the TCA cycle with an increased production of lactate, which can be utilized by *Borrelia* to sustain its metabolism. PBMC shift to anaerobic glycolysis accommodates an increased energy demand, and contributes to the inflammatory response both innate and adaptive immune system [21]. Results from our clinical study have also revealed *Borrelia* CoA-disulfide reductase, which plays a central role to regenerate NAD+ to sustain glycolysis [22]. In relation to carbohydrate metabolisms, urine of symptomatic Borreliosis patients yielded *Borrelia* PTS glucose transporter, which supports the established notion that *Borrelia* is expected to rely primarily on glucose as a carbon source in the mammalian host. The spirochete, however, is considered capable of using other carbon sources including mannose, N-acetylglucosammine, maltose, chitobiose, and glycerol. Our identification of *Borrelia* PTS mannose transporter in patient urine suggests that *Borrelia* might use mannose as an alternative carbon source in the human host [21].

*Borrelia* has limited fatty acid metabolism capabilities. It lacks the ability to synthesize new fatty acids, to extend existing fatty acid chains, and to catabolize fatty acids to derive energy through beta oxidation [21]. The *Borrelia* cellular membrane, however, is rich in fatty acids, mainly palmitate, covalently linked to lipoproteins (outer surface proteins and VlsE). Unusually for a prokaryotic organism, the *Borrelia* membrane also contains polyunsaturated fatty acids, which are likely derived from the host environment. Clinical and preclinical studies show that *Borrelia* infection triggers eicosanoid production in the vertebrate host [21]. Eicosanoid generation is likely mediated by human lipases that cleave arachidonic acid. However, the fact that eicosanoid induction precedes immune cell influx to the site of infection has prompted scientists to postulate a direct interaction between *Borrelia* and host cell membrane lipids. A *Borrelia* lipase like enzyme has been discovered [23], which can act as phospholipase and contribute to eicosanoid release. *Borrelia* lipase detection in the urine of Borreliosis patients in our clinical proteomics study supports the notion that *Borrelia* interacts directly with lipids in host cell membranes to aid the production of unsaturated fatty acids that become assimilated in lipids and lipoproteins incorporated in its cellular envelope. Polyunsaturated fatty acids in the cell envelope are a known target for reactive oxygen species produced by immune cells of the infected host [23].

## Proteomics Reveals Vulnerable Metabolic, Lipid, and Glycolic Features of Borreliosis In Vivo

*Borrelia* has all the homologs of the mevalonate pathway, which leads to the synthesis of isopentyl-5-phopsphate (IPP) and dimethylallyl pyrophosphate (DMAP). However, *Borrelia* is unable to use IPP and DMAP to synthesize cholesterol [21]. Consequently, it has been proposed that IPP and DMAP are used to synthesize peptidoglycans as cell-wall components or possibly for post-translational modification of proteins. Recently, a complete pathway has been proposed in which acetate is the precursor for mevalonate and isoprenoids that are then converted to undecaprenyl phosphate which is essential for cell wall biosynthesis [24]. The importance of the mevalonate pathway is confirmed by the fact that treatment of spirochete cultures with statins, which target the mevalonate pathway, slow *Borrelia* growth [25]. In our clinical study we found *Borrelia* type 2 isopentenyldiphosphate delta-isomerase which belongs to the mevalonate pathway and mediates the synthesis of IPP.

*Borrelia* contains only two main membrane phospholipids: phosphatidylglycerol (PG) and phosphatidylcholine (PC). PG biosynthesis in *Borrelia* proceeds through the enzyme phosphatidylglycerolphosphate synthase (PGs, BB0721), which uses CDP-diacylglycerol and glycerol-3-phosphate as substrate. In our proteomics study, we identified the *Borrelia* enzyme phosphatidate cytidylyltransferase (2.7.7.41), which mediates the synthesis of CDP-diacylglycerol from a-1,2 –diacyl-glycerol 3-phosphate and it is the fourth step in the PG synthesis immediately before PGs [26]. CDP-diacylglycerol synthesized by phosphatidate cytidylyltransferase can also be used by *Borrelia* phosphatidylcholine synthase BB0249 in conjunction with exogenous choline to yield PC, the other major phospholipid in the *Borrelia* membrane [26].

## Further Functional Molecular Insights of *Borrelia* Homeostasis Mechanisms

Our analysis revealed two enzymes involved in the biosynthesis of nicotinamide adenine dinucleotide NAD from nicotinic acid: *Borrelia* nicotinate phosphoribosyltransferase and nicotinate (nicotinamide) nucleotide adenylyltransferase NAD+ homeostasis is vital for many cellular processes, including glycolysis. A *Borrelia* encoded nicotinamidase that converts nicotinamide in nicotinic acid for NAD+ synthesis is essential for bacterial replication and infectivity.

There are no oxidative pathways known to be active in *Borrelia*; thus, no reactive oxygen species (ROS) are expected to be produced endogenously [21]. *Borrelia*, however, is exposed to ROS in the host, during phagocytosis and inflammatory response. Iron mediated, DNA oxidative damage, typical of most bacteria, is limited in *Borrelia* because it has evolved to function with very low levels of intracellular iron [27] and lacks iron binding proteins. Effective DNA repair mechanisms may also help *Borrelia* suffer from limited oxidative damage [21]. We identified a number of enzymes involved in DNA repair in the urine of patients: excinuclease uvra, exodeoxyribonuclease, DNA mismatch repair proteins muts and mutl, DNA protecting protein dpra, endonuclease muts2, DNA primase, DNA polymerase, DNA topoisomerase, and DNA ligase.

*Borrelia* lipid membrane, rich in polyunsaturated fatty acid, is considered an alternative target of ROS-mediated damage [28]. The key enzyme to degrade ROS and limit oxidative damage in *Borrelia* is superoxide dismutase [29], which we found in our proteomics study in the urine of Borreliosis patients. Superoxide dismutase activity was detected in *Borrelia* in 1997 [30] and its activity is essential for virulence. *Borrelia* superoxide dismutase is a metalloenzyme that uses manganese as co-factor [29]. In patient urine, we identified *Borrelia* magnesium transporter MgtE which aids the import of magnesium to support metalloprotein functions. An additional protein found in our clinical study that indirectly protects the spirochete against ROS mediate membrane damage is *Borrelia* CoA-disulfide reductase. CoA-disulfide reductase reduces CoA-disulfides to CoA in an NADH-dependent manner to maintain thiol-disulfide homeostasis [22]. It has been demonstrated that CoA-disulfide reductase is essential for *Borrelia* to establish infection in the human host [22].

*Borrelia* lacks the cellular pathways for *de novo* purine synthesis and is required to salvage purines and pyrimidines from the host environment to synthesize its nucleic acids [31]. In contrast to *Borrelia burgdorferi*, *Borrelia hermsii* and *Borrelia turicatae* have a complete pathway for purine salvage, which is thought to be one of the reasons why relapsing fever spirochetes achieve higher densities in blood [31]. One of the enzymes that have been identified in *B. hermsii* and *B. turicatae* and is absent in *B. burgdorferi* is adenylosuccinate synthase, which was detected in the urinary peptidome of patients in our clinical study. Purine metabolism is essential for *Borrelia* growth and virulence in mammals [31]. *Borrelia burgdorferi* has a distinct pathway for purine salvage [32]. *B. burdgorferi* imports adenine and hypoxanthine from the tick and vertebrate host. In our clinical study, human urine yielded the detection of a *Borrelia* uracil-xanthine permease that imports xanthine and a range of nucleobases that can be catabolized. A series of enzymatic reactions convert hypoxanthine to guanosine monophosphate (*GMP*) and deoxyguanosine monophosphate (*dGMP*), the building block for RNA and DNA synthesis, respectively. The last enzyme in the pathway is glutamine-hydrolyzing GMP synthase (guaA), which we found in our clinical study, GuaA, is essential for *Borrelia* survival in the tick-vertebrate infection cycle [31].

Our clinical study identified *Borrelia* glycoside hydrolase family 3 protein (BB_0620), which is an enzyme involved in sugar metabolism and catalyzes the reaction of chitobiose to N-acetyl-D-glucosamine (Kegg bbu00520). This enzyme has been associated to a pathway of *Borrelia* resistance to beta-lactams. As a consequence of inhibition of peptidoglycan biosynthesis and increase of muropeptides, BB_0620 is thought to participate in the induction of expression of beta-lactamases (Kegg pathways bbu00520, bbu01100, bbu01501).

## Further Verification of the Functional Relevance of Biofluid Proteomics

We further identified *Borrelia* Bax inhibitor-1/YccA family protein a transmembrane protein thought to regulate mammal host cell survival and apoptosis. It has been demonstrated that expression of the gene coding for this protein is implicated in the shift from a cell survival to apoptotic state in neuroblastoma cells co-cultured with *B. burgdorferi* and primary microglia [33].

*Borrelia* Acriflavine resistance protein is a transmembrane protein involved in the resistance-nodulation-division (RND) family of drug efflux systems [34]. A multi drug resistance pump including acriflavine resistance protein has been identified in Borrelia, and it has been shown to be involved in virulence and resistance to antibiotic treatments [34].

*Borrelia turicatae* phage portal protein (A0A172XCQ6) was identified in our clinical study. *B. burgdorferi* bacteriophages have been identified and partially characterized [35]. Prophage systems have been identified in the *Borrelia* burgdorferi genome [36]. Bacterial phage systems can facilitate genome rearrangement, laterally transfer genetic material between *Borrelia* strains, introduce new virulence or fitness factors, or lyse competing strains through prophage induction [37]. A circular plasmids of 32 kb in *Borrelia burgdorferi* encodes, among other proteins, for a BBL01, Putative bacteriophage portal protein. Portal proteins are incorporated in the growing phage and are required for DNA packaging. Other bacteria (*Haemophilus influenzae*, *Xylella fastidiosa*, *Salmonella enterica* serovar Typhi, and *Enterococcus faecalis*) carry putative bacteriophage portal proteins [37]. Previous studies demonstrated that BBL01 was downregulated when *Borrelia burgdorferi* cultivated *in vitro* was exposed to blood, suggesting that *B. burgdorferi* expresses BBL01 in the tick host preferentially, while mammal host conditions induce a suppression of the protein; protein suppression is confirmed by the fact that BBL01 antibody response was not detected in *B. burgdorferi* infected mice [37]. Our detection of a *Borrelia turicatae* phage portal protein in human urine, if confirmed, might suggest that *B. turicatae* has a prophage system, and that the *B. turicatae* differential expression of phage derived proteins in the invertebrate and vertebrate host is different than *B. burgdorferi’s*.

Thus, proteomic analysis of urine from symptomatic patients, reveals a long list of functional molecular insights about the physiologic state of the pathogen at the time of specimen collection, and generates further insights into vulnerabilities of *Borrelia* that can be targeted for future treatment strategies.

## Source and Dissemination Mechanisms of Urinary Borreliosis Biomarkers

**Figure 2:**
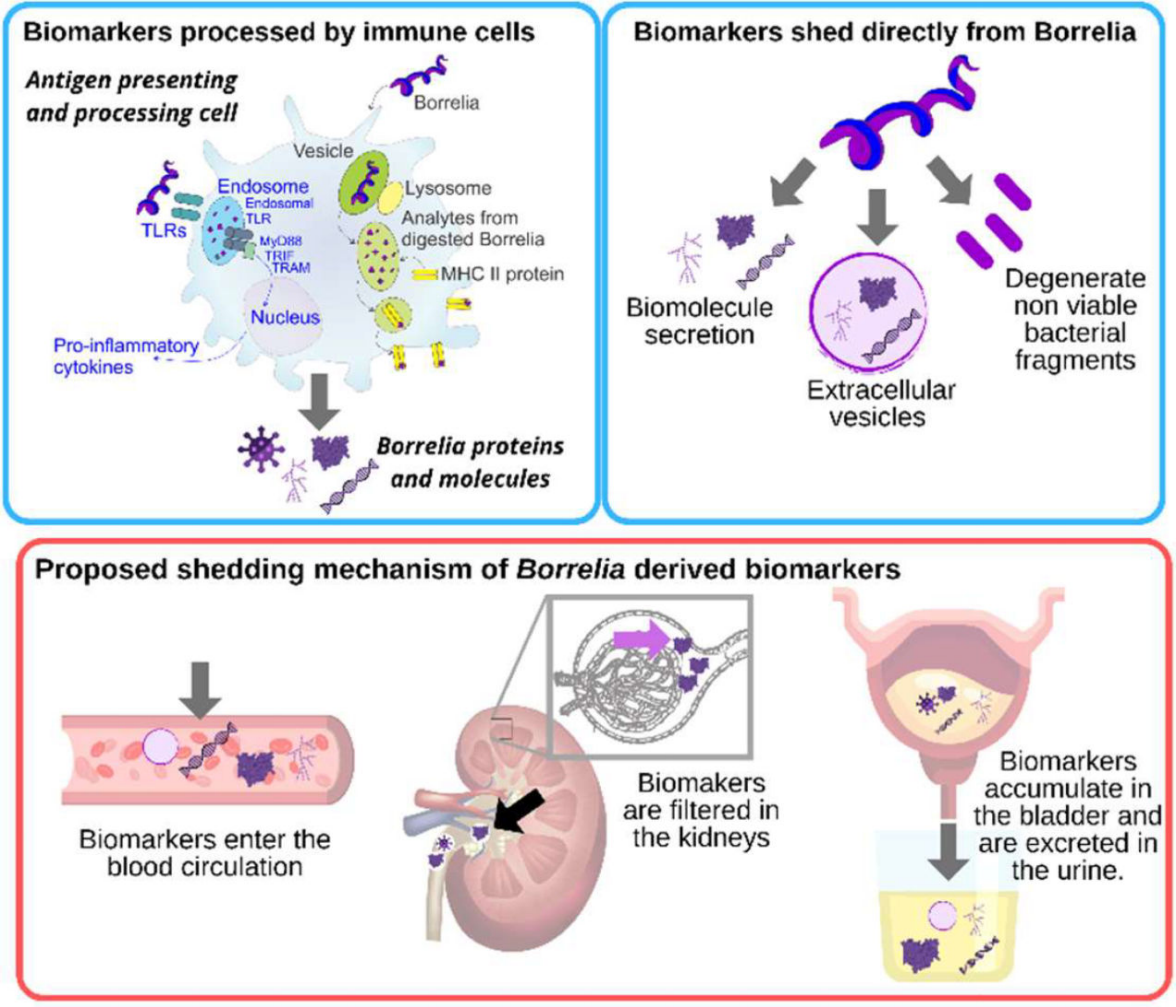
Proposed sources and shedding mechanisms of urinary, *Borrelia*-derived biomarkers.

Similarly, to other pathogens (*Trypanosoma cruzi* [38], *Mycobacterium tuberculosis* [39,40], *Toxoplasma gondii* [41]), two possible sources of *Borrelia* derived peptides and protein fragments detected in peripheral fluids have been proposed [20]: a) spirochete peptides generated from the interaction of host immune cells, such as antigen presenting cells, with the pathogen, and b) peptides that derive directly from viable or non-viable spirochetes (Figure 2). Bacterial cell wall fragments have been previously detected in a rat model of chronic Lyme Borreliosis up to 360 days after spirochete challenge [42]. Persistence of *Borrelia* peptidoglycan in the synovial environment of patients suffering from Lyme arthritis was detected for several weeks after antibiotic treatment [19]. It has been shown that live *Borrelia* spirochetes can disrupt the architecture of lymph nodes, and impede the formation of stable germinal centers [43]. Animal studies highlight another peculiarity in *Borrelia* immunology: generation of anti-*Borrelia* neutralizing antibodies is MHC class II independent [43]. It has been suggested that *Borrelia* primed dendritic cells undergo cytolysis mediated by natural killer cells, thus releasing their antigenic content [43]. Experimental data on Lyme arthritis patients and animal models support the notion that synovial fibroblasts can act as non-professional phagocytic and antigen presenting cells. Fibroblasts primed with *Borrelia* expressed MHC class II molecules and effector cytokines involved in lymphocyte activation. Culture studies demonstrated that primary mouse fibroblasts were able to internalize *Borrelia* [44]. The peripheral fluid concentration of *Borrelia* antigens and peptides derived from immune cells is unknown and presumably exceedingly low. This supports the use of highly sensitive analytical technologies, and of pre-analytical sample processing steps aimed at concentrating low abundance analytes. A urinary *Borrelia* peptide identified in our clinical study was compatible with MHC II presentation thus supporting the involvement of immune cells in its shedding [20].

The second source of *Borrelia* peptides can be direct shedding by spirochetes present in different tissues (e.g. synovial connective tissue [45], brain [46], and heart [47]). Proteins and biomolecules are secreted by *Borrelia* in a soluble, vesicle free form [48] and vesicle-bound [49,50]. Non-viable bacterial fragments can be an additional source of systemic *Borrelia* peptides and bioanalytes [51] (Figure 2).

*Borrelia* derived peptides, similarly to cancer derived biomarkers, can enter blood circulation by passively penetrating blood vessel walls [52]. Blood circulating biomarkers undergo glomerular filtration and tubular readsorption in the kidneys, are concentrated in the bladder, and eventually are excreted in the urine [53]. (Figure 2). Biomarkers are thought to reside in the blood for a short period of time due to homeostatic regulation. Peripheral biomarkers eliminated from blood are integrated over time in the urine [52]. Thus urine appears to have advantages for tick-borne disease diagnostics for several reasons, in addition to the large repertoire of pathogen specific peptides found in our clinical study. Urine integrates a circulating low concentration analyte cleared in the kidneys over time, such that the total number of analyte molecules in the entire urine volume is much greater than those present in a spot blood sample. Urine testing is also non-invasive and can be easily conducted longitudinally following diagnosis and treatment of a patient presenting with a tick bite. Longitudinal testing for *Borrelia* derived molecular analytes offers a definitive means of distinguishing whether or not persistent symptoms are associated with persistent presence of the pathogen itself (Figure 1).

## Conclusions

Sensitive and accurate direct tests can adjudicate active pathogen infection and contribute to solving long-standing controversies about the etiology of posttreatment persistent symptoms causing great suffering for Borrieliosis patients. High throughput technologies such as nextGen sequencing and proteomics are promising approaches for a direct test that is insensitive to strain variability. We have shown that proteomics can provide functional information that has the potential to contribute to our understanding of pathogen behavior in the human host and can complement information provided by nucleic acid amplification based techniques. High throughput highly sensitive technologies need to be validated longitudinally on controlled cohorts of well characterized Borreliosis patients, before and after antibiotic therapies.
